# Pickering Emulsion-Based Gels with Halloysite as a Stabilizer: Formulation, Mechanical Properties and In Vitro Drug Release Studies

**DOI:** 10.3390/molecules30051087

**Published:** 2025-02-27

**Authors:** Anna Froelich

**Affiliations:** 3D Printing Division, Chair and Department of Pharmaceutical Technology, Poznan University of Medical Sciences, 3 Rokietnicka Street, 60-806 Poznań, Poland; froelich@ump.edu.pl

**Keywords:** Pickering emulsion, halloysite, lidocaine, poloxamer, nanotubes, rheology, texture profile analysis, Franz diffusion cells

## Abstract

Lidocaine is an analgesic agent frequently incorporated in topical formulations intended for application in minor surgical procedures or relieving neuropathic pain associated with numerous conditions, including post-herpetic neuralgia or diabetic peripheral neuropathy. In this study, Pickering o/w emulsions with halloysite nanotubes as a stabilizing agent and lidocaine incorporated in the internal phase were formulated with the use of the Quality by Design (QbD) approach. The selected emulsions were transformed into semisolid gels with poloxamer 407 as a thickening agent, and investigated for rheological and textural properties, indicating the mechanical features of the obtained gels. Moreover, the obtained formulations were tested for lidocaine release with the use of vertical Franz diffusion cells in order to assess the relationship between the applied composition and potential clinical applicability of the analyzed gels. The obtained results indicate that the emulsion droplet diameter is affected mostly by the oil and halloysite contents. The yield stress points, hardness and cohesiveness values of the obtained gels increased with the oil content. The drug release rate seems to be affected mostly by the concentration of the active ingredient in the oil phase.

## 1. Introduction

Pickering emulsions are known as dispersion systems composed of two immiscible phases revealing different polarities stabilized with solid particles localized at the interfacial surface. The solid stabilizer plays the same role as a conventional chemical surfactant applied in a classical emulsion. Even though the first scientific reports regarding these systems were published in the beginning of the 20th century [1], their potential in numerous scientific and industrial areas was recognized relatively late with the development of materials science leading to the acquisition of novel particles suitable for emulsion stabilizing [2]. Since then, the potential applications of Pickering emulsions have been investigated in numerous fields, including the food industry [3], personal care products [4], oil recovery [5], pharmaceutical technology [6] and many others [7,8]. The described systems are also investigated as templates for the formation of capsules via reinforcement of emulsion-stabilizing particles with various techniques [9]. In the area of drug delivery, Pickering emulsions are taken into consideration as potential oral [10] and topical [11] dosage forms. As lipid-based formulations containing both low- and high-polarity phases, they offer numerous advantages as drug carriers, particularly in the case of poorly water-soluble active agents including the substances belonging to Biopharmaceutics Classification System (BCS) classes II and IV [12]. As formulations designed for dermal administration, they are suitable for both hydrophilic [13] and lipophilic [14] actives, depending on the emulsion type. One of the most important advantages related to the topical application of Pickering emulsions is the lack of chemical surfactants. The conventional surface-active agents are known for their irritancy properties towards the skin [15], and contact dermatitis caused by the action of topically administered surfactants is well-characterized in the scientific literature [16,17,18]. On the other hand, surfactants may also affect the pharmacokinetics of the active ingredients [19]. Pickering emulsions are surfactant-free which makes them an interesting alternative to classical emulsions, both in drug delivery and personal care products formulation.

A crucial element of Pickering emulsions are solid particles adsorbed at the interface of the immiscible phases and mechanically preventing the coalescence of emulsion droplets. As stabilizing agents, silica, clays, calcium carbonate, titanium dioxide and many others have been investigated. The most recent studies focus on novel organic particles designed to obtain emulsions displaying specific properties, for example the ability to change physicochemical properties upon contact with some external factors, like temperature or pH change. These smart systems are regarded as potentially useful in oil recovery, catalysis and biomedical sciences, including in drug delivery [20]. On the other hand, an increased demand for non-toxic, biocompatible and biodegradable materials results in the intensification of research on particles of natural origin, including polysaccharide- or protein-based particles. The mentioned stabilizers are particularly useful in Pickering emulsions investigated for food and personal care applications [21].

**Figure 1:**
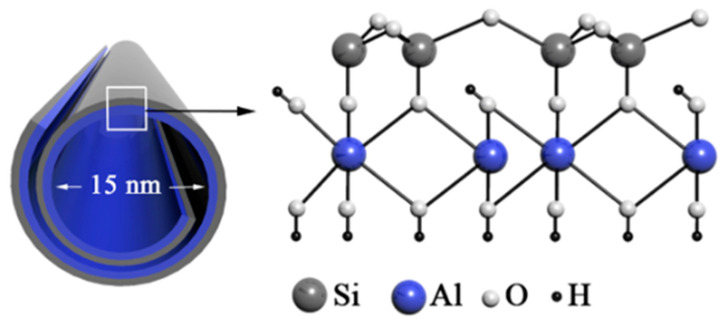
Structure and morphology of halloysite nanotubes. Adapted with permission from [25].

Halloysite [Al_2_Si_2_O_5_(OH)_4_·nH_2_O; n = 0–2] is a natural aluminosilicate clay occurring in a nanotubular form, with a tetrahedral silicate layer located at the external surface of nanotubes and octahedral layers of Al_2_O_3_ exposed to the internal lumen of nanotubes and the inner spaces between the adjacent aluminosilicate layers [22]. The inner and outer diameters of halloysite nanotube are 10–20 and 50 nm, respectively, and its length ranges from 200 to 1500 nm [23]. Depending on the hydration state, the spacing between layers can adopt values of 7 and 10 Å for the dehydrated and hydrated forms, respectively. The tube wall consists of 10–15 aluminosilicate layers rolled together into a cylinder [24] (Figure 1). It is noteworthy that halloysite nanotubes have different electrostatic charges deposited in the lumen and at the outer surface. The external surface is charged negatively, while the internal aluminol surface is charged positively which allows for binding cationic and anionic substances outside and inside the nanotube, respectively [20].

It is noteworthy that it is considered to be a relatively non-toxic and biocompatible nanomaterial which was shown in the studies involving zebrafish embryos performed by Long et al. [23] and cell cultures investigated by Vergaro et al. [26]. The toxicity of nanotubes and other nanomaterials extensively investigated in various scientific and industrial areas is a major concern, as indicated in numerous reports [27,28]. In drug delivery research, halloysite is frequently investigated as a carrier for controlled drug release formulations. It is important to note that it can effectively bind multiple active ingredients, revealing different polarities which is related to the varied properties of the external and internal surface of nanotubes which can be further modulated with the use of different pH or additional agents, like polyvinyl alcohol, poly(lactic-co-glycolic acid), polyacrylates, polysaccharides and many others. The interaction with macromolecular agents leads to stronger binding of the active pharmaceutical ingredients and sustained release. In liquid systems composed of two immiscible phases with different polarities, halloysite nanotubes can act as stabilizers adsorbed at oil–water interface which was already reported in the literature [29,30]. However, most of the available reports are related to industrial and environmental applications including oil spill remediation [31], restoration of works of art [32], as well as obtaining novel porous materials [33]. Halloysite-based Pickering emulsions in drug delivery have been utilized only as templates for obtaining novel drug-loaded microspheres [34] and antibacterial agent-loaded coatings for silk sutures [35]. Even though Pickering emulsions have gained scientific interest as potential drug delivery systems for dermal applications [36], no studies regarding halloysite-based systems have been reported so far.

Lidocaine (2-(diethylamino)-N-(2,6-dimethylphenyl)acetamide) is commonly known as a local anesthetic agent, frequently applied in minor surgical procedures since its introduction to the pharmaceutical market in the 1940s [37]. It acts through non-selective blocking of voltage-gated channels and stabilization of neuronal membranes. The current research indicates that its action is much more complex and may involve interactions with a number of other receptors and molecular mechanisms further contributing to its efficacy [38]. Since 1999, it has also been accepted by the Food and Drug Administration (FDA) in the management of postherpetic neuralgia [39]. The most recent studies and meta-analyses indicate that dermal formulations with lidocaine may be useful in the treatment of many other conditions associated with pain, including diabetic peripheral neuropathy, carpal tunnel syndrome and osteoarthritis [37,39,40].

In this study, halloysite-based oil-in-water emulsions with pharmaceutically acceptable excipients loaded with lidocaine, a model local anesthetic drug revealing poor solubility in water, are obtained and characterized. It is noteworthy that Pickering emulsion-based systems can be an interesting alternative to conventional surfactant-based emulsions, particularly in dermal drug delivery. It must be emphasized that, even though the subject of emulsions stabilized with solid nanoparticles is not new, the studies investigating formulations of potential applicability as topical drug delivery systems are quite scarce. On the other hand, halloysite applied in this study can act not only as an emulsion stabilizer but is also interesting in terms of possible drug incorporation. Considering the fact that liquid pharmaceutical formulations can be regarded as less convenient for dermal applications than semisolid ones, poloxamer 407 was applied as a thickening agent. Transformation of liquid Pickering emulsions into gels was also applied to prevent the creaming reported in other studies [29,41]. The composition of emulsions was optimized, and the obtained gels were tested for rheological and textural properties, as well as drug release. The main purpose of the study was to obtain a novel complex semisolid system with Pickering emulsion and to evaluate its basic physicochemical and pharmaceutical properties important in terms of potential topical application. The aim of the planned experiments was to investigate the relationship between the composition of halloysite-based Pickering emulsions and the properties of lidocaine-based gels with special attention paid to the features affecting potential therapeutic efficacy. The described semisolid formulations could be considered as an alternative for the conventional dosage forms loaded with poorly water-soluble drugs intended to be administered to the skin surface.

## 2. Results and Discussion

### 2.1. Variable Screening

**Figure 2:**
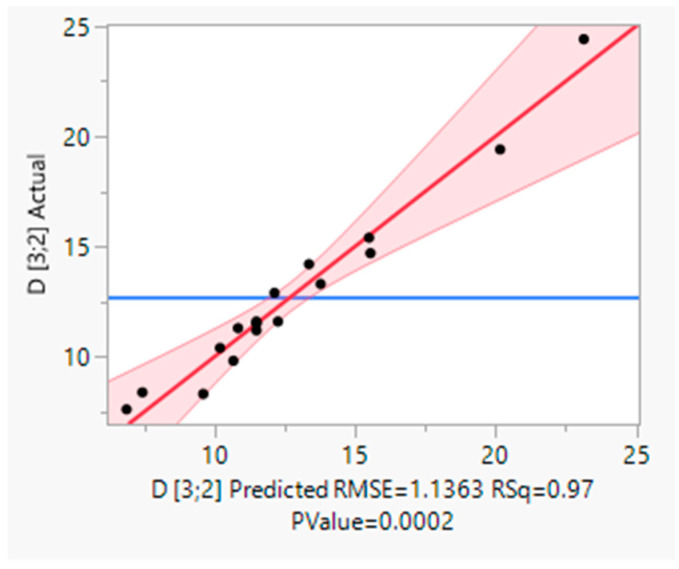
Actual (black dots) vs. predicted (red line) values plot. The red area corresponds to the 95% confidence region.

**Figure 3:**
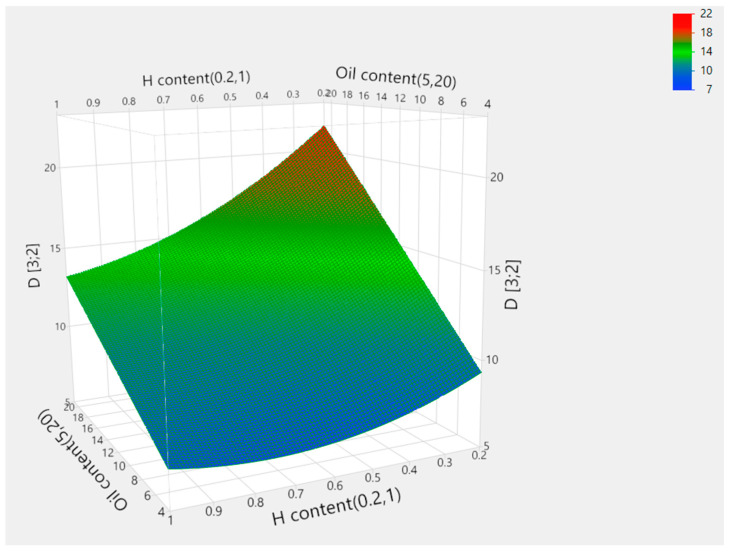
The 3D response of Sauter mean diameter (D [3;2]) to oil and halloysite (H) content changes.

The parameters of the emulsification process (oil concentration, halloysite concentration and homogenization time), and the obtained Sauter mean diameter values measured for the analyzed samples and predicted with the use of central composite design (CCD) procedure are summarized in Table 1. The predictions were made with the use of the optimized model described by Equation (1). The correlation plot showing the relationship between the experimental and predicted data is summarized in Figure 2. In Table 2, the effects of the applied variables, including two-way interactions and quadratic effects, are depicted in an ascending order corresponding to their significance. Taking into consideration the obtained *p* values it may be assumed that in the performed procedure, oil and halloysite content had the highest significance, while the homogenization time did not affect droplet diameter in the obtained emulsions. Moreover, there is a two-way interaction between the two statistically significant parameters which means that the effect exerted by one of them is dependent on the other one. For the remaining interactions no statistical significance was observed, as calculated *p* values exceeded 0.05. Figure 3 illustrates the effects of oil and halloysite contents on droplet diameter, depicted with the use of 3D response surface methodology. In general, lower Sauter mean diameter values were associated with higher halloysite and lower oil contents which is a quite obvious result, regarding the roles of the particular emulsion components. Similar effects regarding the oil [42,43] and solid particles content [44] are reported by other researchers. However, in our study, the response to the changes in solid stabilizer content is nonlinear. Interestingly, Kempin and Drews [45] conducted a study involving a similar emulsification technique which investigated in detail the impact of the parameters, including shear rate, on droplet diameter. According to the results obtained, increasing the homogenizer tip speed resulted in a decrease in droplet diameter. However, it must be emphasized that in the cited work a different emulsion composition was applied and the range of analyzed shear rates was wider.

(1) $$d_{3,2}=9.53+0.70X_{1}-9.16X_{2}-0.27X_{3}-1.25\cdot 10^{-6}X_{1}^{2}+8.47X_{2}^{2}+0.01X_{3}^{2}-0.46X_{1}X_{2}+0.02X_{1}X_{3}+0.23X_{2}X_{3}$$

**Figure 4:**
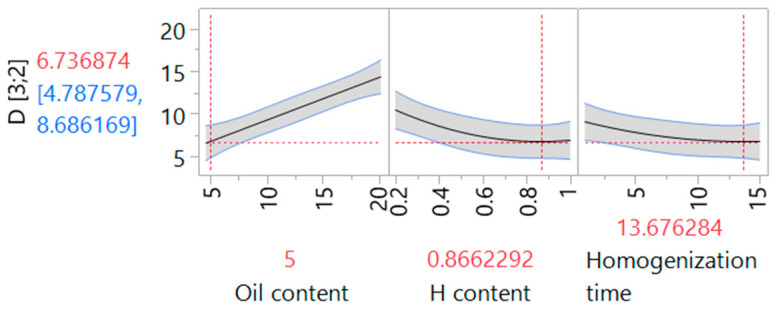
Prediction profilers of Sauter mean diameter (D [3;2]) as a function of oil and halloysite (H) content changes and homogenization time. The red numbers and red dotted lines indicate the predicted value (vertical axis) and optimized parameters (horizontal axes), while the gray areas, blue lines and blue numbers show confidence intervals.

Figure 4 illustrates prediction profilers showing optimized oil and halloysite contents with homogenization time with the aim of obtaining the smallest possible droplet diameter. The optimized parameters with predicted and experimental values are summarized in Table 3. It may be noted that the measured value lies within the theoretical boundaries depicted as a gray area in Figure 4. It is noteworthy that similar systems obtained with the use of halloysite nanotubes are described in the literature. Stehl et al. [29] described Pickering emulsions obtained with the use of 1-dodecene as an oil phase and different concentrations of halloysite ranging from 0.05 to 1.0%. In this study, the Sauter diameter changes are categorized into three regions, with an initial decrease at a low nanoparticle concentration, a further increase between 0.1 and 0.2%, and a gradual decrease at higher concentrations. The observed changes were ascribed to different halloysite packing at the surface of the emulsion droplets. A similar trend was observed in the 3D response surface depicted in Figure 3; however, the Sauter mean values obtained in our study were lower. This effect could be related to the differences in the applied oil phase and higher oil content applied in the literature study. Similarly, droplet diameter observed by Cavallaro et al. [46] for halloysite-stabilized emulsions with n-decane ranged from 20 to 40 μm. Again, higher droplet diameter could be related to different oil phase and oil content, as well as different emulsification conditions. In this case, the systems were obtained by 2 min of vortex mixing and the oil phase content was significantly higher which can correlate with the observed differences.

### 2.2. Gel Formation and Characterization

**Figure 5:**
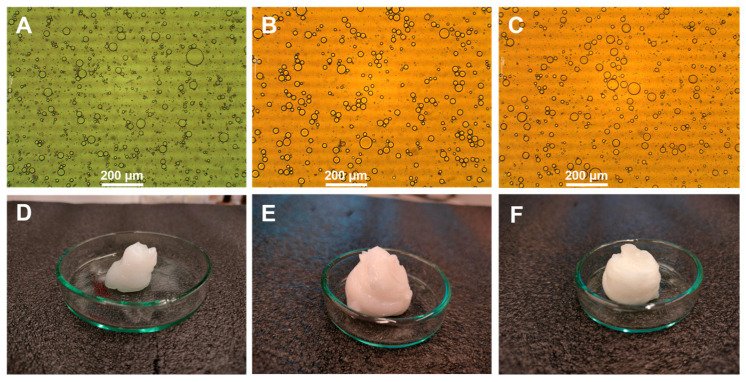
Optical microscopy images of the emulsions used in further studies ((**A**): emulsion 5, (**B**): emulsion 12, (**C**): emulsion 16) and the corresponding placebo gels ((**D**): G5, (**E**): G12, (**F**): G16).

The parameter screening described above showed that the droplet diameter depends mostly on the oil and solid stabilizer content, while the impact of the homogenization time seemed to be statistically insignificant. In the next step of the study, the selected emulsions (emulsions 5, 12 and 16, Table 1) were transformed into gels in order to check the impact of the emulsion composition on the properties of the resulting semisolid system. The emulsions selected for further studies contained different amounts of oil, while halloysite content and the thickening polymer contents were kept at the same level. As it was already mentioned by other researchers [47], emulsion viscosity can be affected by the oil content. It may be expected that transforming emulsions displaying different concentrations of a low-polarity phase may lead to semisolid gels also differing in terms of physicochemical properties, which can further affect the drug release rate. Moreover, the application of different oil contents can contribute to textural and rheological differences between gels. The mechanical properties are of particular importance in topical dosage forms because they determine the way the product is perceived by the patient. The optical microscopy images of the emulsions used to obtain gels with the images depicting placebo gels are presented in Figure 5.

#### 2.2.1. Rheological Studies

**Figure 6:**
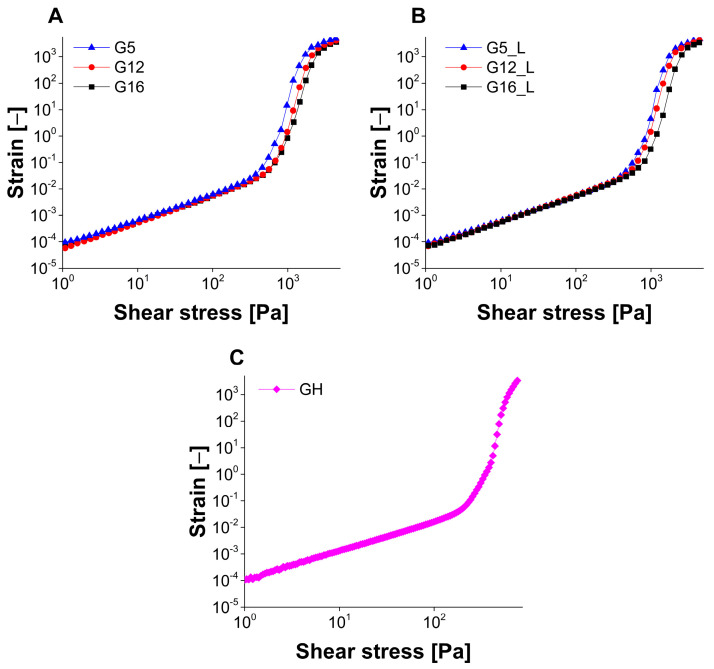
The results of rotational rheological experiments performed in controlled stress mode for placebo (**A**), lidocaine-loaded gels (**B**) and reference halloysite-loaded gel (**C**).

**Figure 7:**
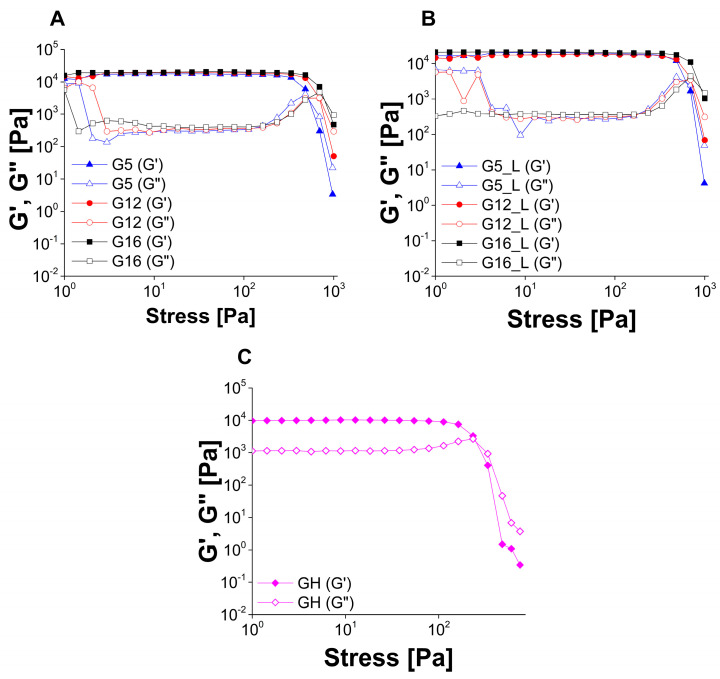
The results of oscillatory stress sweep experiments performed for placebo (**A**), lidocaine-loaded gels (**B**) and reference halloysite-loaded gel (**C**).

**Figure 8:**
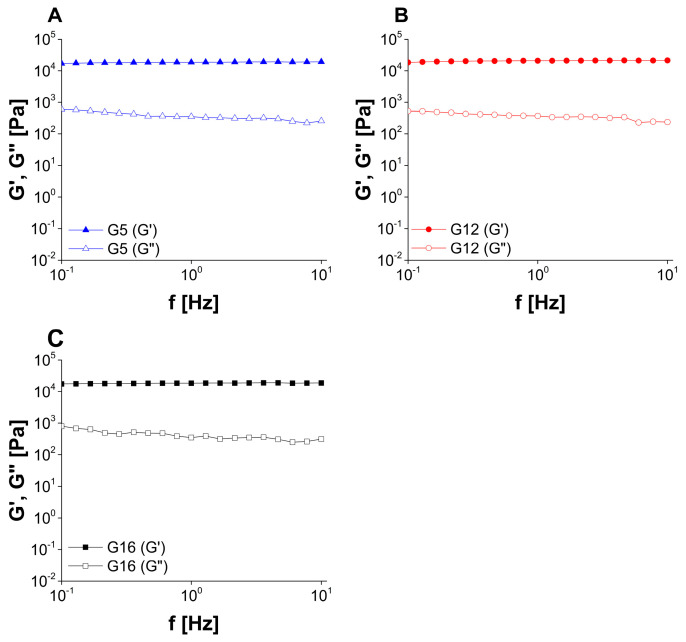
The results of oscillatory frequency sweep experiments performed for placebo gels ((**A**): G5, (**B**): G12, (**C**): G16).

The results of controlled stress and oscillatory experiments are depicted in Figure 5, Figure 6, Figure 7 and Figure 8, while the yield stress values calculated from the intersection of the lines obtained from the extrapolation of approximately linear sections of the curve (controlled stress tests) and from storage and loss moduli crossover points (oscillatory stress sweep tests) are summarized in Table 4. The flow curves obtained in the rotational experiments (Figure 5) are typical for non-Newtonian, shear-thinning systems, including weak physical gels and similar results were already described in the literature for 20% (*w*/*w*) poloxamer and composite poloxamer/alginate gels, with flow behavior dependent on the composition of the investigated gels [48]. The results of the study performed for Pickering emulsion-loaded gels indicate that the recorded yield stress values were affected by the composition of the emulsion. In both placebo and lidocaine-loaded gels the recorded points increased with the increase in oil content. As it was already mentioned in the scientific literature regarding gels with incorporated emulsions [49,50,51], the dispersed phase droplets can behave differently depending on the possible interactions between the emulsion stabilizer deposited at the oil–water interface and the surrounding polymer matrix. In so-called emulsion-filled gels, oil droplets can act either as active or inactive fillers, either weakening or strengthening the gel structure. On the other hand, particulate gels can be formed by clustering emulsion droplets aggregating into a three-dimensional network.

The consistency and strength of the investigated gels are clearly affected by the presence of emulsion droplets. Taking into consideration the mentioned models describing the structure of emulsion-loaded gels, it may be assumed that Pickering emulsion droplets are an active component of the system and interact with the polymer chains forming the gel network. For the comparative analysis, reference gel containing only halloysite dispersed in the water phase was prepared and investigated. It is noteworthy that the yield stress value observed in the case of the reference gel was significantly lower than those recorded for the emulsion-loaded systems. Moreover, the obtained strain–stress relationship was different (Figure 5), indicating a double yielding phenomenon which was not seen in the samples with incorporated emulsion. According to Ahuja et al. [52], numerous different materials can display this behavior, including gels and suspensions. In systems with suspended particles, it is usually associated with the presence of interconnected aggregates and a two-step process involving the disruption of the network in the first stage and further breaking the aggregates [53]. However, poloxamer 407 gels also display yield points [54] and the double yielding phenomenon can also occur as an overlapping effect caused by the presence of hydrogel matrix and halloysite agglomerates.

The results of oscillatory stress sweep tests generally confirmed the conclusions drawn from the rotational experiments. The yield stress points measured with this method increased with larger oil content and lidocaine-loaded gels had higher yield stress values than the corresponding placebo gels (Figure 7, Table 4). However, the conclusions regarding double yielding phenomenon in the case of halloysite-loaded gel analyzed with oscillatory stress sweep test (Figure 7C) are ambiguous, as only slight deviations in the storage and loss moduli curves are observed at the end of the experimental range.

**Figure 9:**
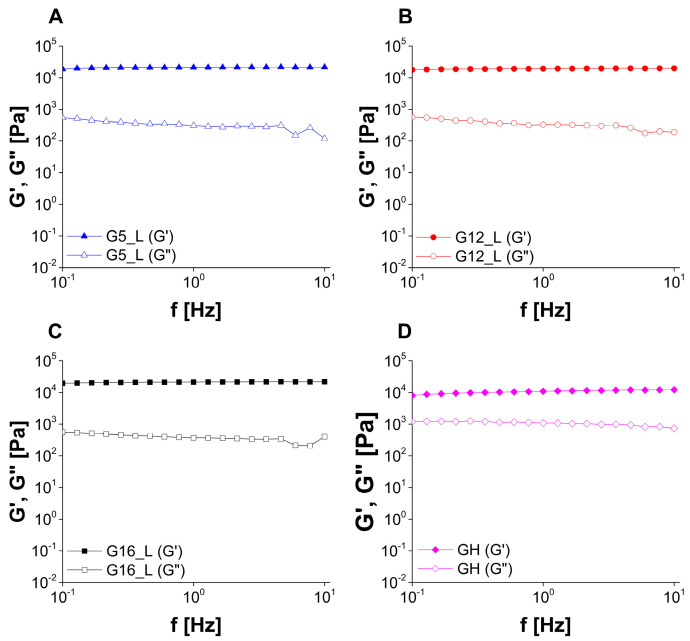
The results of oscillatory frequency sweep experiments performed for lidocaine-loaded gels ((**A**): G5_L, (**B**): G12_L, (**C**): G16_L) and reference halloysite-loaded gel (**D**).

Taking into consideration the results of frequency sweep tests (Figure 8 and Figure 9) and the rheological classification system proposed by Ross-Murphy [55,56], it may be concluded that all of the investigated samples can be classified as weak physical gels, as storage modulus (G′) was slightly higher than loss modulus (G″) and both parameters revealed only slight dependence on the frequency. The observed behavior corresponded to the properties of poloxamer 407 gels investigated above gelation temperature [57]. In weak gels the structure is formed by weak non-covalent interactions, including hydrogen bonds and van der Waals interactions, and these interactions can be easily broken as a result of a shear stress.

#### 2.2.2. Texture Profile Analysis (TPA)

**Figure 10:**
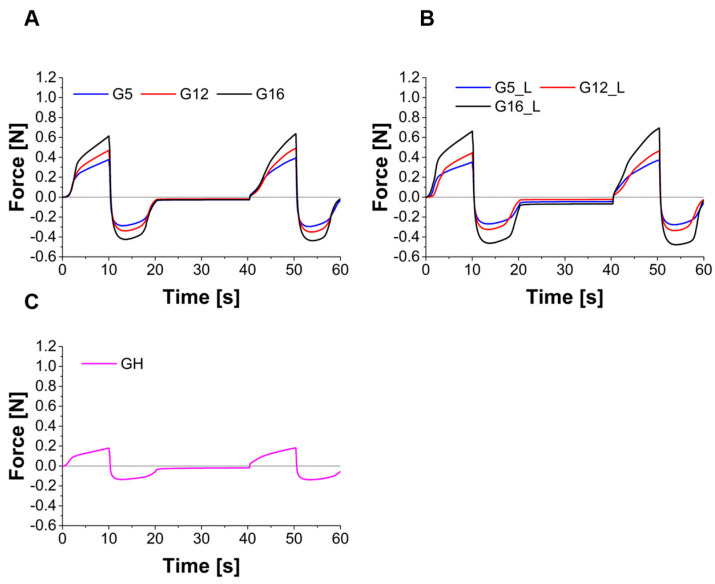
The results of texture profile analysis performed for placebo gels (**A**), lidocaine-loaded gels (**B**) and reference halloysite-loaded gel (**C**).

The force vs. time curves obtained in TPA tests are presented in Figure 10, while the calculated textural parameters are summarized in Table 5. Textural studies have been frequently employed to characterize sensory properties of various food products to investigate their behavior during chewing or in the manufacturing process [58]. Recently, some of the techniques used in food analysis have been adopted in pharmaceutical studies, particularly those focusing on the description of the mechanical properties of semisolid dosage forms [59,60,61]. The textural parameters, including hardness, cohesiveness and adhesiveness, are important in terms of product development and applicability, especially in dermal formulations. As already mentioned by other authors [62], ease of product removal from the container and retention time on the skin are of particular importance in such cases.

The statistical analyses performed for the textural data indicate that the sample hardness depended on the oil content both in the placebo and the drug-loaded groups. In the placebo group, cohesiveness is higher for the gel with the smallest oil content (G5), while for gels G12 and G16 no statistically significant differences were observed. In the lidocaine-loaded group, the sample with the highest oil content (G16_L) displayed the lowest cohesiveness, while the gels with lower oil contents were similar to each other. In both groups, the absolute values of the adhesiveness parameter were significantly higher in the samples with higher oil content (G16 and G16_L, respectively). The impact of lidocaine presence on the textural parameters is ambiguous, as in the samples with the lowest oil content (G5 and G5_L) no statistically significant difference is observed for any parameter, while for an intermediate oil content the cohesiveness is higher for the sample with lidocaine (G12_L) and in the samples with the highest oil content hardness and adhesiveness are higher in the presence of the drug.

**Figure 11:**
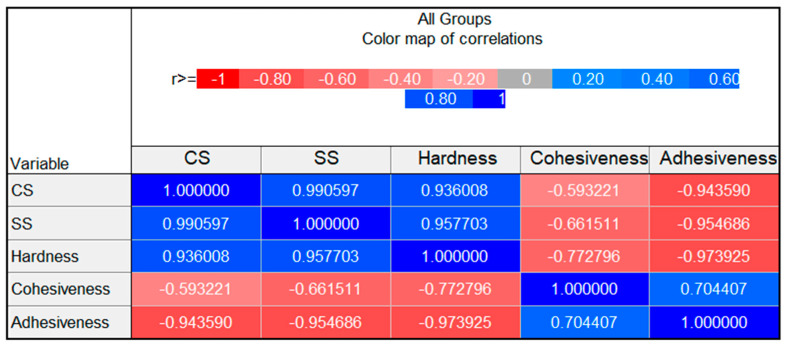
The correlation matrix depicting the relationships between yield stress points obtained in CS and SS modes, hardness, cohesiveness and adhesiveness (1 corresponds to ideal positive correlation, while −1 corresponds to ideal negative correlation).

In Figure 11, the correlation matrix depicting the relationship between the investigated mechanical parameters is shown. The values of Pearson’s r coefficient summarized in the matrix indicate a strong positive [63,64] correlation between yield stress values obtained with different methods, which is quite obvious, and also a strong positive correlation between both yield stress parameters and hardness. Strong negative correlations are observed between adhesiveness and hardness, as well as between adhesiveness and both yield stress points, which is just a matter of annotation and can be explained by the negative values of adhesiveness. In fact, the samples revealing higher hardness and higher yield stress values are also more adhesive. The cohesiveness parameter seems to be negatively correlated with hardness and yield points and positively correlated with the adhesiveness parameter, however, the correlation is moderate. In all analyzed combinations, the correlations were statistically significant.

#### 2.2.3. Drug Release Studies

**Figure 12:**
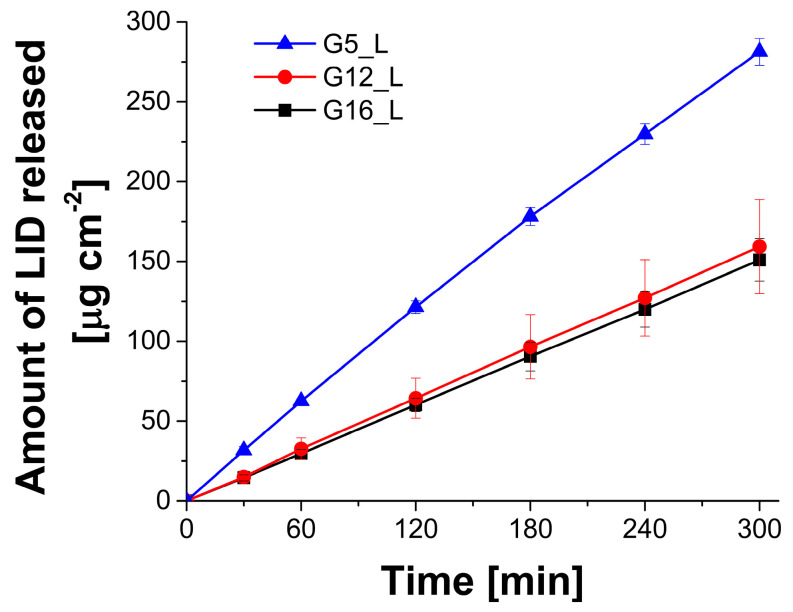
The results of drug release experiments performed for lidocaine-loaded gels G5_L, G12_L and G16_L (n = 5).

In the last step of the study, the lidocaine-loaded gels (G5_L, G12_L and G16_L) were subjected to drug release tests with the use of vertical Franz diffusion cells widely employed in the analyses focusing on the formulations intended for dermal applications [65,66]. It is important to notice that drug release investigations provide valuable information on the rate of diffusion of the active ingredient from the formulation to the acceptor medium. Moreover, the obtained results can be used in comparative analyses whenever the impact of the formulation composition on its potential efficacy is taken into consideration. However, it must be emphasized that these studies are conducted with the use of simplified models involving only synthetic membranes and any simulation of the biological effects related to the interaction of the formulation components with the skin structures cannot be performed. Therefore, the further steps of dermal formulations should comprise experiments employing ex vivo animal or human skin models [67,68]. The results of the tests shown as the cumulated amount of the released lidocaine vs. time are depicted in Figure 12.

It can be assumed that the gel composition affected the drug release rate. In all investigated formulations the drug release profiles are linear, with the highest slope observed for G5_L, containing the lowest concentration of the oil phase. Formulations G12_L and G16_L displayed similar properties and lidocaine release rate was lower in the case of these two gels. The flux values with R^2^ values calculated for the analyzed gels are summarized in Table 6.

**Figure 13:**
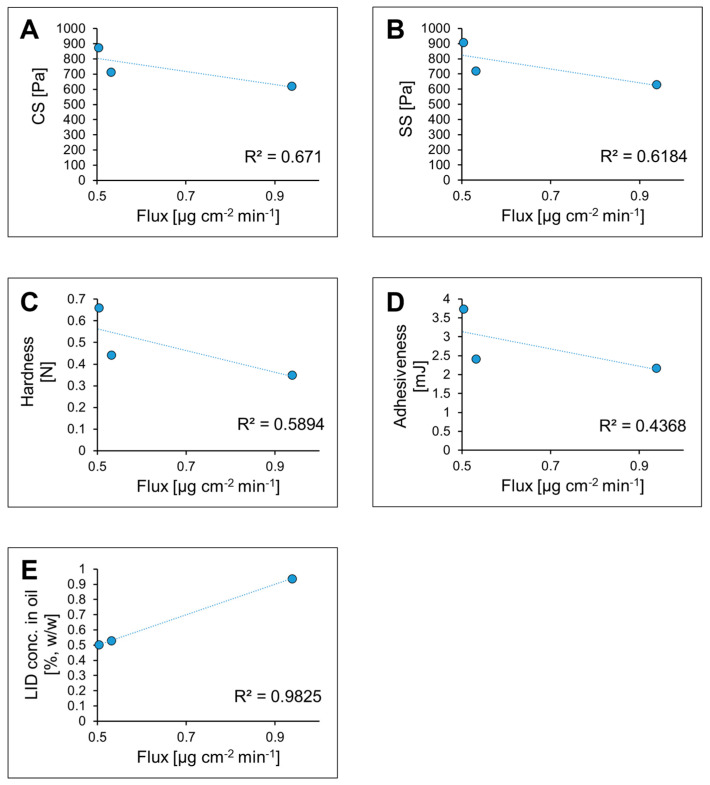
The analysis of correlations between the mechanical parameters ((**A**): yield stress point obtained in CS experiments; (**B**): yield stress point obtained in oscillatory studies; (**C**): hardness; (**D**): adhesiveness) and LID concentration in the oil phase (**E**) and the calculated flux values. In plot (**D**), absolute values of adhesiveness are presented.

The differences between the analyzed lidocaine-loaded gels observed in the drug release experiment might be quite surprising, considering the same lidocaine content in all formulations and statistically significant differences was recorded for all samples in the rheological and textural studies. Taking into account the results of all the mechanical studies described above, it may be assumed that hardness and yield stress values, reflecting the general structure strength, are increasing with the increase in the oil phase content. However, the drug release rate is higher only for the sample with the lowest concentration of ethyl oleate, while no statistically significant differences are observed for the gels with intermediate and the highest oil content (G12_L and G16_L, respectively). The graphic representation of the relationship between the calculated flux values and the measured rheological and textural parameters indicates no simple linear correlation and the R^2^ values are relatively low (Figure 13A–D). Nevertheless, it must be emphasized that the lidocaine base applied in the study reveals low solubility in water and good solubility in the oil phase and this phenomenon should be kept in mind whenever the drug diffusion is taken into consideration. The theoretical concentration of LID in the oil phases in the investigated gels are 25.3, 10.1, and 6.3% (*w*/*w*) for G5_L, G12_L and G16_L, respectively. The plot depicted in Figure 13E showing the correlation between the concentration of the drug and the observed flux indicates that the quantitative relationship between the hydrophobic drug and the oil phase seems to be the most important factor affecting the diffusion rate, which is most probably related to the gradient between the internal phase of the emulsion and the acceptor fluid.

## 3. Materials and Methods

### 3.1. Materials

Halloysite nanotubes (H), ethyl oleate (EO), Kolliphor P407 (poloxamer 407; P407) were obtained from Sigma-Aldrich Poland (Poznań, Poland) and used as received. Lidocaine base (LID) was purchased from Pol-Aura (Poznań, Poland). In all performed experiments deionized water purified with Simplicity^®^ Water Purification System (Merck Millipore, Burlington, MA, USA) was used. Acetonitrile (HPLC-grade; ACN) was purchased from Avantor Performance Materials Poland S. A. (Gliwice, Poland).

### 3.2. Emulsion Formation and Characterization

In the first step, halloysite nanotubes (H) were dispersed in deionized water to obtain 1.0% (*w*/*w*) suspension. The desired amounts of water and H were weighed and placed in a bottle. The suspension was sonicated for 30 min with the use of an ultrasonic bath and after that the bottle was closed and the mixture was stirred with magnetic stirrer for 24 h at 500 rpm.

Emulsion was obtained with the use of high-shear homogenization method performed with UltraTurrax T25 (IKA, Staufen, Germany) homogenizer equipped with S25N-10G (IKA, Staufen, Germany) homogenizing tip. The desired amounts of oil and halloysite suspension were placed in 25 mL beaker and mixed with magnetic stirrer at 500 rpm for 1 min. Next, the initial emulsion was homogenized at 11,000 rpm for a predetermined time.

#### 3.2.1. Variable Screening

The significance of different variables and their interactions was checked with experimental design procedure. All calculations were done with JMP^®^ Pro16.0.0 (SAS Institute Inc., Cary, NC, USA). In order to estimate the impact of the selected independent variables on the properties of Pickering emulsions, three parameters (X_1_, X_2_, X_3_) at three levels each were analyzed (Table 7). As a response, emulsion droplet diameter defined as Sauter mean (d_3,2_; µm) (Equation (2)) [69] was investigated. For the analysis, the central composite design procedure was employed and 17 experimental runs were generated and randomized with JMP^®^ Pro software, ver. 18.0.2 (SAS Institute, Cary, NC, USA).

(2) $$d_{3,2}=\frac{\sum n_{i}d_{i}^{3}}{\sum n_{i}d_{i}^{2}}$$

where $n_{i}$ is a number of particles displaying diameter $d_{i}$.

#### 3.2.2. Droplet Diameter Measurements

Pickering emulsions droplet diameter was measured with Mastersizer 3000 (Malvern Instruments Ltd., Malvern, UK) equipped with Hydro SV unit and red and blue light sources operating at 632.8 and 470 nm, respectively. In each experiment, the unit was filled with 6.0 mL of deionized water and then 20 μL of emulsion was added. The diluted emulsion was stirred at a rate of 1500 rpm in order to avoid multiple scattering effects. The size distribution was calculated with the use of Mie theory and the refractive indices of 1.332 and 1.451 were used for water and ethyl oleate, respectively [70]. For each emulsion, five subsequent measurements were performed and average values were calculated.

#### 3.2.3. Optical Microscopy Imaging

The optical microscopy images of the selected emulsions were obtained at a magnification of 100× with B3 Professional Series (Motic, Xiamen, China) optical microscope equipped with Digital Moticam 2300 camera and a computer with Motic Images Plus 2.0 software (Motic, Xiamen, China).

### 3.3. Gel Formation and Characterization

Poloxamer-based gels were obtained with the use of “hot” method [71]. The composition of the gel samples is presented in Table 8. For samples G5, G12 and G16, with and without lidocaine emulsions, 5, 12 and 16 (Table 1) were used, respectively. Poloxamer 407 was weighed and added to the emulsion and the mixture was placed in a water bath at 80.0 ± 0.5 °C and stirred at 800 rpm for 4 h until the polymer was completely dissolved. After this time, the mixture was stirred further at room temperature until the gel was formed.

#### 3.3.1. Rheological Studies

All measurements were performed at 25.0 ± 0.5 °C with a HAAKE™ Rheostress1 rotational rheometer (ThermoFisher Scientific, Waltham, MA, USA) equipped with parallel 35 mm diameter plate geometry (PP35Ti, gap size: 1 mm) and a temperature-controlled unit Thermo HAAKE™ DC30. Each test was performed in triplicate and for each run a fresh gel sample was applied. The obtained rheological data were recorded and processed with HAAKE™ RheoWin™ software, ver. 3.40.00 (ThermoFisher Scientific, Waltham, MA, USA).

##### Flow Behavior and Yield Stress Studies

Flow behavior of the investigated gels was analyzed in controlled stress (CS) mode. The shear stress increased from 1 to 5000 Pa in 15 s, except for the GH gel which was analyzed with shear stress increasing from 1 to 500 Pa in 30 s. As a result, strain vs. stress curves were obtained and the yield stress values were obtained as intersection points of tangential lines fitted to the two approximately linear parts of the curve.

##### Oscillatory Studies

In the first step, the samples were subjected to oscillatory stress sweep tests. The oscillatory stress increased from 1 to 1000 Pa at a constant frequency of 1.0 Hz. Storage and loss moduli (G′ and G″, respectively) were plotted as a function of oscillatory stress to estimate the linear viscoelasticity region (LVR) and select the value of oscillatory stress necessary to perform oscillatory frequency tests. Moreover, intersection points of G′ and G″ vs. oscillatory stress curves were recorded.

Oscillatory frequency studies were performed at the amplitude of 10 Pa (sample G16) or 50 Pa (all other gels) with a frequency ranging from 0.1 to 10.0 Hz. The results were recorded as G′ and G″ vs. oscillatory frequency curves.

#### 3.3.2. Texture Profile Analysis (TPA)

The textural studies were performed with the use of Autograph AGS-X universal tester (Shimadzu, Kyoto, Japan) equipped with a 10 N loading cell and steel cylindrical probe with a 10 mm diameter. The samples (15.0 g) were placed in 25 mL beakers and compressed twice with the probe velocity of 60 mm min^−1^. In each compression cycle the probe was immersed in the sample to a depth of 10 mm and the interval time between the cycles was 20 s. Each test was performed in triplicate at an ambient temperature. The data were obtained and processed with TrapeziumX software ver. 1.5.2 (Shimadzu, Kyoto, Japan). From the resulting force vs. time curves, textural parameters including hardness, adhesiveness, cohesiveness and gel strength were calculated. The definitions of textural parameters are presented elsewhere [72].

#### 3.3.3. Drug Release Studies

The lidocaine release study was performed with the use of vertical Franz diffusion cells (PermeGear, Hellertown, PA, USA) equipped with regenerated cellulose membranes (Visking^®^ dialysis tubing, SERVA Electrophoresis GmbH, Heidelberg, Germany) characterized by a 12,000–14,000 molecular weight cut off (MWCO) value and ca. 25 Å pore diameter. The effective diffusion area of the cells was 0.999 cm^2^. Each cell was filled with 8.0 mL of acceptor fluid consisting of a phosphate buffer and ethanol mixture (80:20, *v*/*v*). In each cell (n = 5), 1.0 mL of the gel was placed on the upper surface of the membrane in the donor compartment and the donor compartment was sealed with parafilm to prevent water evaporation during the experiment. The study was performed at 32.0 ± 0.5 °C, with receptor fluid stirred at 200 rpm. The samples (0.2 mL) were withdrawn from the acceptor compartment after 30, 60, 120, 180, 240 and 300 min from gel application. The withdrawn sample was immediately replaced with an equal volume of the receptor fluid and the concentration of the drug in the samples was quantified with the use of a validated high-performance liquid chromatography (HPLC) method. The cumulative amounts of released drug per unit area (Q) were calculated according to Equation (3) [73].

(3) $$Q=\frac{C_{n}\cdot V+\sum_{i=1}^{n-1}C_{i}\cdot S}{A}$$

where $C_{n}$ is a the concentration of lidocaine obtained at nth timepoint [μg mL^−1^], V is Franz cell volume [mL], $\sum_{i=1}^{n-1}C_{i}$ is a sum of the drug concentrations determined at timepoints 1 through n−1 [μg mL^−1^], S is sample volume [mL] and A is the surface of the membrane [cm^2^].

HPLC analysis was performed with Nexera-i-LC-2040C 3D (Shimadzu, Kyoto, Japan) UHPLC system equipped with Hypersil GOLD™ C18 column (250 mm × 4.6 mm, 5 μm; Thermo Fisher Scientific, Waltham, MA, USA). The analytical wavelength was set at 230 nm. The tests were performed at isocratic conditions with a mobile phase flow rate of 2.0 mL min^−1^. The mobile phase consisted of a monopotassium phosphate solution (4.85 g L^−1^) adjusted to pH = 8.0 with concentrated NaOH solution and acetonitrile (50:50, *v*/*v*). The temperature of the column oven was 30.0 ± 0.1 °C. The injection volume was 20 μL.

### 3.4. Statistical Analysis

The results obtained for different semisolid samples were tested with one-way analysis of variance (ANOVA) followed with post-hoc Scheffe’s test. For the analysis of the correlations between the mechanical parameters, Pearson’s r coefficients were calculated. In all tests the statistical significance level was set at 5%. All calculations were conducted with Statistica software ver. 13.0 (StatSoft, Tulsa, OK, USA).

## 4. Conclusions

In this study, novel Pickering emulsions with halloysite nanotubes as a stabilizing agent were obtained and the formulation composition and preparation process were optimized to define the factors affecting particle diameter in the final product. Among the investigated parameters, comprising oil and halloysite concentrations and also the homogenization time, the first two had a statistically significant impact on the obtained emulsion characteristics, while the emulsification time was not significant in the analyzed range. The Sauter mean values ranged from 7.62 to 24.4 μm and lower values were associated with lower oil and higher solid stabilizer contents.

Three of the investigated emulsions differing in terms of oil content were used as liquid media for polymer gel preparation. The performed rheological analyses revealed that the investigated systems were non-Newtonian and shear-thinning, which is typical for poloxamer 407-based systems. The yield stress points obtained from rotational experiments performed in the controlled stress mode, as well as from oscillatory studies, increased with the increase of oil content. It was also found that lidocaine-loaded gels had higher yield points than the corresponding placebo gels. The reference gel containing halloysite particles dispersed in a poloxamer gel matrix without an oil phase revealed significantly lower yield point values which may indicate that the emulsion droplets interact with the polymer chains network contributing to the gel strength. The results of oscillatory rheological studies show that in the analyzed samples storage modulus (G′) prevails over the loss modulus (G″) and the samples can be classified as weak physical gels.

Hardness and adhesiveness determined in texture profile analysis can be generally correlated with the yield stress points, as their values increase with oil content. However, in this case the impact of the drug’s presence is not clear. Cohesiveness of the tested gels seems to be moderately correlated with the previously discussed parameters.

In the drug release study, higher lidocaine diffusion rates were observed for the gel with the lowest oil content, revealing also the lowest hardness, adhesiveness and yield points values. However, the gels with intermediate and high oil content did not differ in terms of their ability to release the active ingredient, even though the systems differed significantly in terms of the mechanical parameters. Taking into consideration the hydrophobic properties of the lidocaine base applied in the study, it may be hypothesized that the most important parameter defining the drug release rate in this case is the concentration of the active ingredient in an oil phase, as a linear relationship between these two parameters was observed.

## Figures and Tables

**Table 1:** Emulsification process parameters with the results of particle size analysis and predicted values (X_1_, X_2_ and X_3_ are oil concentration, halloysite concentration and homogenization time, respectively).

ID	Pattern	X_1_ [%, *w*/*w*]	X_2_ [%, *w*/*w*]	X_3_ [min]	d_3,2_ (exp.) [μm]	d_3,2_ (pred.) [μm]
1	− − −	5.0	0.2	1	11.3 ± 0.3	10.8
2	− − +	5.0	0.2	15	9.82 ± 0.09	10.7
3	a 0 0	5.0	0.6	8	8.39 ± 0.12	7.4
4	− + −	5.0	1.0	1	8.32 ± 0.09	9.6
5	− + +	5.0	1.0	15	7.62 ± 0.11	6.9
6	0 a 0	12.5	0.2	8	15.4 ± 0.2	15.5
7	0 0 a	12.5	0.6	1	12.9 ± 0.1	12.1
8	0 0 0	12.5	0.6	8	11.2 ± 0.2	11.5
9	0 0 0	12.5	0.6	8	11.6 ± 0.1	11.5
10	0 0 0	12.5	0.6	8	11.5 ± 0.1	11.5
11	0 0 A	12.5	0.6	15	11.6 ± 0.1	12.3
12	0 A 0	12.5	1.0	8	10.4 ± 0.1	10.2
13	+ − −	20.0	0.2	1	19.4 ± 0.3	20.2
14	+ − +	20.0	0.2	15	24.4 ± 0.3	23.1
15	A 0 0	20.0	0.6	8	14.7 ± 0.5	15.6
16	+ + −	20.0	1.0	1	14.2 ± 0.2	13.4
17	+ + +	20.0	1.0	15	13.3 ± 0.1	13.8

**Table 2:** The effects of the applied variables. The bars depict logworth value, while the blue dashed line corresponds to *p* = 0.01 (logworth = 2).

Source	Logworth		*p* Value
X_1_ (5,20)	5.018	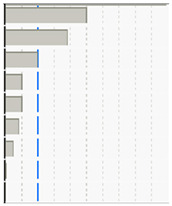	0.00001
X_2_ (0.2,1)	3.814		0.00015
X_1_·X_2_	1.977		0.01055
X_1_·X_3_	1.038		0.09163
X_2_·X_2_	1.037		0.09193
X_2_·X_3_	0.809		0.15518
X_3_·X_3_	0.464		0.34371
X_3_ (1,15)	0.062		0.86790
X_1_·X_1_	0.000		0.99992

**Table 3:** Optimized parameters with predicted and experimental d_3,2_ values.

Optimized Parameters	d_3,2_ [μm]	
	Predicted	Experimental
Oil content: 5.0% (*w*/*w*)	6.73 ± 1.95	7.85 ± 0.09
H content: 0.86% (*w*/*w*)		
Homogenization time: 13.68 min		

**Table 4:** Yield stress points obtained in controlled stress (CS) and oscillatory stress sweep (SS) tests.

Sample	Yield Stress (CS) [Pa]	Crossover Point (SS) [Pa]
G5	537.5 ± 9.3	546.0 ± 3.7
G12	653.4 ± 4.3	685.0 ± 2.9
G16	732.1 ± 7.8	810.2 ± 2.8
G5_L	620.7 ± 6.6	631.1 ± 9.3
G12_L	715.4 ± 11.8	721.0 ± 8.9
G16_L	874.1 ± 12.7	908.0 ± 6.8
GH	I: 203.7 ± 13.6 II: 398.8 ± 5.8	269.2 ± 1.1

**Table 5:** Textural parameter obtained for the investigated samples.

Sample	Hardness [N]	Cohesiveness [—]	Adhesiveness [mJ]
G5	0.376 ± 0.010	1.027 ± 0.018	−2.222 ± 0.168
G12	0.469 ± 0.018	0.978 ± 0.019	−2.497 ± 0.209
G16	0.612 ± 0.016	0.967 ± 0.010	−3.115 ± 0.079
G5_L	0.351 ± 0.007	1.040 ± 0.010	−2.179 ± 0.053
G12_L	0.443 ± 0.015	1.028 ± 0.013	−2.415 ± 0.082
G16_L	0.660 ± 0.008	0.988 ± 0.011	−3.738 ± 0.215
GH	0.179 ± 0.003	1.043 ± 0.006	−1.122 ± 0.039

**Table 6:** Flux and R^2^ values calculated for the lidocaine-loaded gels.

Sample	Flux [μg cm^−2^ min^−1^]	R^2^ [−]
G5_L	0.938 ± 0.027	0.9986 ± 0.0003
G12_L	0.531 ± 0.099	0.9992 ± 0.0002
G16_L	0.503 ± 0.045	0.9993 ± 0.0005

**Table 7:** Factors screened and the experimental domain.

Symbols	Independent Variables	Levels		
		Low (−1)	Medium (0)	High (+1)
X_1_	Oil content [%, *w*/*w*]	5.0	12.5	20.0
X_2_	Halloysite content [%, *w*/*w*]	0.2	0.6	1.0
X_3_	Homogenization time [min]	1	8	15

**Table 8:** Composition of gel samples.

ID	Emulsion ID *	Emulsion Content [%, *w*/*w*]	Lidocaine [%, *w*/*w*]	Poloxamer 407 [%, *w*/*w*]
G5	5	80.0	—	20.00
G12	12	80.0	—	20.00
G16	16	80.0	—	20.00
G5_L	5	79.0	1.0	20.00
G12_L	12	79.0	1.0	20.00
G16_L	16	79.0	1.0	20.00

* According to Table 1.

## Data Availability

The experimental data is available upon the direct request.

## Abbreviations

BCS	Biopharmaceutics Classification System
FDA	United States Food and Drug Administration
CCD	Central Composite Design
H	Halloysite
G′	Storage modulus
G″	Loss modulus
CS	Controlled stress rotational test
SS	Stress sweep oscillatory test
EO	Ethyl oleate
LID	Lidocaine
LVR	Linear viscoelasticity region
TPA	Texture profile analysis
MWCO	Molecular weight cut-off
HPLC	High-performance liquid chromatography
ANOVA	Analysis of variance
